# Identification of CD8+ T Cell-Related Genes: Correlations with Immune Phenotypes and Outcomes of Liver Cancer

**DOI:** 10.1155/2021/9960905

**Published:** 2021-06-02

**Authors:** Qi Pan, Ying Cheng, Donghua Cheng

**Affiliations:** Department of Hepatobiliary Surgery and Organ Transplantation, Key Laboratory of Organ Transplantation of Liaoning Province, First Hospital of China Medical University, China

## Abstract

**Purpose:**

Treatment outcomes for advanced liver cancer are poor. Immunotherapy is a treatment strategy that has been widely used to treat other cancers. Studies have shown that CD8+ T lymphocytes are essential factors affecting the efficacy of immunotherapy. We used computational biology methods to determine the coexpressed gene network that promotes CD8+ T lymphocyte infiltration.

**Method:**

We obtained the liver cancer gene matrix and clinical follow-up information data from TCGA liver hepatocellular carcinoma FPKM. We obtained single nucleotide polymorphism (SNP) data to evaluate the tumor mutation burden. The “estimate” package and the CIBERSORT algorithm were used to evaluate tumor purity and the proportion of CD8+ T lymphocytes in the liver cancer cohort. We used the gene expression matrix of liver cancer and the relative proportion of CD8+ T lymphocytes as input files and performed WGCNA based on this analysis. The weighted coexpression network identified the most CD8+ T lymphocyte-related coexpression modules in liver cancer. Then, we analyzed the biological processes involved in the module. We determined the coexpression module with CD8+ T lymphocyte infiltration in terms of data and function. We then screened the factors in the coexpression module correlated with CD8+ T lymphocyte content greater than 0.4. Finally, the expression levels of these factors were verified at the protein level using immunohistochemistry and single-cell sequencing.

**Results:**

We determined the CD8+ T lymphocyte proportions that correlated with coexpression networks. Four coexpressed genes (C1QC, CD3D, GZMA, and PSMB9) were identified as CD8+ T cell coexpression genes that promoted infiltration of CD8+ T cells. Because the factors in the coexpression network often participate in similar biological processes, we found that these factors were most related to antigen processing and presentation of peptide antigen through functional enrichment. In the clinical phenotype analysis, we found that 18 factors can be used as independent prognostic protective factors. We found that these factors were significantly negatively correlated with tumor purity and negatively correlated with M2 macrophages in the immunophenotyping analysis. Using immunohistochemistry and single-cell sequencing analysis, we found that CD3D antibody staining was weaker in tumor tissues than normal tissues and was related to CD8+ T cells.

**Conclusion:**

These coexpressed genes were positively related to the high infiltration proportion of CD8+ T lymphocytes in an antigen presentation process. The biological process might provide new directions for patients who are insensitive to immune therapy.

## 1. Introduction

In recent years, breakthroughs have been made in using immune checkpoint inhibitors [1], ushering in a new era to treat advanced tumors. The emergence of immunotherapy provides options for the treatment of liver cancer; these include immune checkpoint inhibitors, adoptive cell transfer, tumor vaccines, and cytokine therapy. Immune checkpoint inhibitors enhance antitumor immune responses by reversing the exhaustion of T cell function and restoring immune recognition and immune attack. Immune checkpoint inhibitor targets include programmed death-ligand 1 (PD-L1) and its receptor PD-1 (programmed cell death protein 1) and cytotoxic T lymphocyte-related antigen 4. PD-1 is a member of the CD28 family and is expressed on the surface of most immune cells, mainly on CD8+ T cells [2]. It binds to PD-L1 and PD-L2 to cause inhibitory signals to be transmitted to T cells and induce tolerance [3]. PD-L1 is abnormally expressed in various tumors, including liver cancer; tumor cells achieve immune escape by abnormally expressing PD-L1 or PD-L2 [4]. Nivolumab (PD-1 monoclonal antibody) [5], pembrolizumab (PD-1 monoclonal antibody), and atezolizumab (PD-L1 monoclonal antibody) have been used to treat advanced liver cancer. Although immunotherapy has achieved gratifying results and has been approved as a treatment option for liver cancer, the objective response rate of the PD-1/PD-L1 antibody alone rarely exceeds 40%. The objective response rate of nivolumab and pembrolizumab in liver cancer was not significantly effective [6]. It is currently believed that the main reason for the low objective response rate of immunotherapy is the emergence of drug resistance [7]. The drug resistance of immunotherapy is a complex and multimechanism interdependent dynamic process, explained by impaired immune infiltration in tumors, depletion of T cells, or recruitment of immunosuppressive cells.

Studies have shown that low PD-1 expression in tumor tissues and low CD8+ T lymphocyte infiltration can cause this insensitivity [8]. According to the results of a meta-analysis of non-small-cell lung cancer, increased numbers of CD8+ tumor-infiltrating lymphocytes are associated with better overall survival [9]. In patients with advanced melanoma treated with pembrolizumab, the densities of CD8+ T cells in the invasion margin and tumor center of the tissue specimens of responders were higher than those of nonresponders [10].

Therefore, this article intends to identify the coexpression modules and functions related to the content of CD8+ T lymphocytes in the tumor microenvironment and define the coexpression network related to the content of CD8+ T cells, to provide a basis for improving the efficacy of immunotherapy.

## 2. Methods

### 2.1. Data Collection and Preprocessing

TCGA expression matrix data from LIHC samples were downloaded from The Cancer Genome Atlas (http://cancergenome.nih.gov/). Tumor transcriptomic profiles of 19530 mRNA were measured in 377 liver hepatocellular carcinoma patients and were brought into the subsequent analysis. The hepatocellular carcinoma single-cell mice sequencing cohort GSE129516 [11] was also downloaded from the GEO database, whose platform is GPL24247.

### 2.2. CD8+ T Cell Proportion Evaluation

To obtain the relative proportion of CD8+ T lymphocytes in each liver hepatocellular carcinoma sample, we used the CIBERSORT [12] method. CIBERSORT evaluated the proportion of 22 tumor-infiltrating immune cells in each sample. The samples with *p* < 0.05 were brought into the WGCNA.

### 2.3. Tumor Microenvironment Score and Tumor Mutation Burden (TMB)

The estimation of stromal and immune cells in malignant tumor tissues [13] is a method that evaluates the proportion of stromal and immune cells by gene expression signatures [13]. To perform a correlation analysis with the tumor mutation burden [14, 15], we obtained TCGA-LIHC SNP data and evaluated the tumor mutation burden of each sample.

### 2.4. Weighted Gene Coexpression Network Analysis (WGCNA)

We used WGCNA [16] to explore the correlations between gene expression and CD8+ T lymphocytes. First, we matched the mRNA matrix of TCGA-LIHC and the corresponding CD8+ T lymphocyte content of the sample and included them in the subsequent WGCNA. To ensure the nonscale of the network, we built a scale-free topology network [17, 18] and set the soft threshold at 5 (*R*‐squared = 0.78, slope = −1.93) and the number of genes in the minimum module at 30.

### 2.5. Gene Ontology Functional Analysis

Gene ontology (GO) [19] analysis was performed to show the biological processes and molecular functions based on the different modules. In this study, we performed GO analysis based on the clusterProfiler [20] package in R3.6.2.

### 2.6. Cox Hazard Proportion Regression Model and Subgroup Evaluation

We applied the Cox risk proportional regression model for the factors in the grey module. The prognostic risk model related to CD8+ T lymphocytes in liver cancer was determined using the forward screening method. We used survival analysis and the area under the curve (AUC) to evaluate the accuracy of the prognostic risk model after rain. Next, we divided TCGA-LIHC cohort into various subgroups according to age, gender, clinical stage, and tumor purity and evaluated the CD8+ T lymphocyte-related risk models in various subgroups.

### 2.7. Gene Set Enrichment Analysis

Gene set enrichment analysis (GSEA) [21] was used to calculate the most involved pathway to these coexpression genes.

### 2.8. Immunohistochemistry

The extracted human tissues were fixed with a 4% formaldehyde buffer. Deparaffinized specimens were then sectioned into 4 *μ*m slices. Tissue slices were incubated at 60°C for 2 h before dewaxing; the sections were autoclaved at 115°C for 3 min for antigen retrieval in a citric acid buffer (pH 6.0) and quenched for endogenous peroxidase activity with 0.3% H_2_O_2_ solution for 15 min. Then, the slices were blocked for nonspecific binding with normal goat serum for 45 min and incubated with the specific primary antibody against C1QC, GZMA, CD3D, and PSMB9 (dilution 1 : 200) overnight at 4°C. Subsequently, the sections were treated with the goat anti-mouse secondary antibody for 30 min at room temperature. Protein expression was visualized using 3,3′-diaminobenzidine. Images were captured using a Nikon Eclipse 80i microscope (Nikon Corporation).

## 3. Results

**Figure 1:**
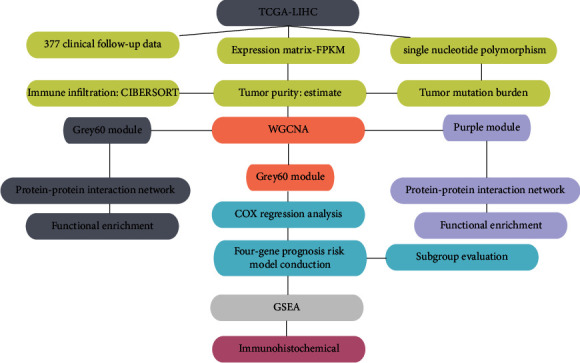
Flow chart of this paper.

A flow chart is displayed in Figure 1, which illustrates the logic of our analysis.

### 3.1. CD8+ T Lymphocyte, Tumor Purity, and Tumor Mutation Burden Evaluation

We measured the CD8+ T lymphocyte proportion, tumor purity, matrix score, immune score, and tumor mutation burden of each LIHC sample. Using the screening principle of *p* < 0.05, we obtained 377 liver cancer samples accurately evaluated by CD8+ T lymphocytes. By integrating the immune-related file with TCGA-LIHC mRNA expression files, we determined WGCNA phenotype entry files.

### 3.2. CD8+ T Lymphocyte Coexpression Network Conduction in TCGA-LIHC

**Figure 2:**
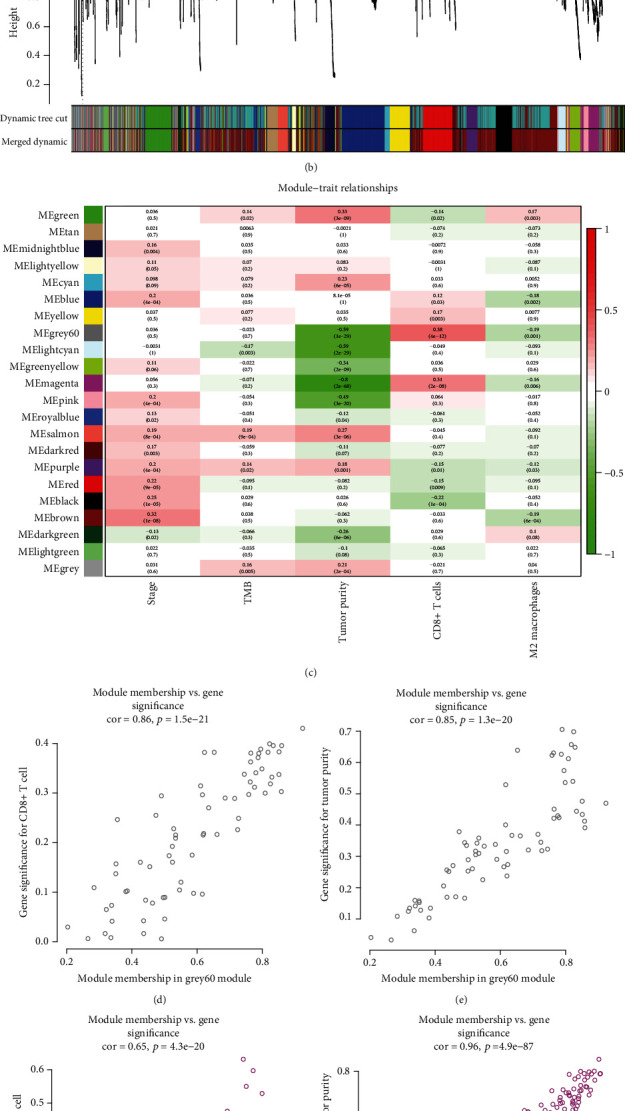
WGCNA. (a) TCGA-LIHC sample clustering results. (b) Twenty-two coexpression modules were conducted by WGCNA. (c) Correlation heat map among different coexpression modules and clinical phenotypes and CD8+ T levels. (d, e) The correlation of genes in grey60 modules with CD8+ T cells and tumor purity. (f, g) The correlation of genes in magenta modules with CD8+ T cells and tumor purity.

WGCNA was performed on TCGA liver hepatocellular carcinomas. To construct a CD8+ T lymphocyte coexpression network, we used a dynamic hybrid cutting method to construct a hierarchical clustering tree (Figure 2(a)). Each leaf on the tree represents a gene, and each branch represents a coexpression module. A total of 22 expression modules were obtained (Figure 2(b)). Then, we calculated the correlation coefficient between each module and CD8+ T lymphocytes and selected the grey60 and purple modules according to the correlation coefficient (Figure 2(c)). The grey60 module had the strongest correlation with CD8+ T lymphocyte proportion in TCGA-LIHC cohort (cor = 0.38; *p* = 4*e* − 12) (Figure 2(c)). The magenta module correlated with the CD8+ T lymphocyte proportion in TCGA-LIHC cohort (cor = 0.31; *p* = 2*e* − 08) (Figure 2(c)). Based on these findings, we supplemented the heat map of the correlation between the factors in the grey60 and magenta modules (Figures 2(d)–2(g)). The grey60 module showed a significant correlation with CD8+ T cells (cor = 0.86, *p* = 1.5*e* − 21) and tumor purity (cor = 0.85, *p* = 1.3*e* − 20). The magenta module showed a significant correlation with CD8+ T cells (cor = 0.65, *p* = 4.3*e* − 20) and tumor purity (cor = 0.96, *p* = 4.9*e* − 87).

### 3.3. CD8+ T Lymphocyte Coexpression Module Functional Analysis

**Figure 3:**
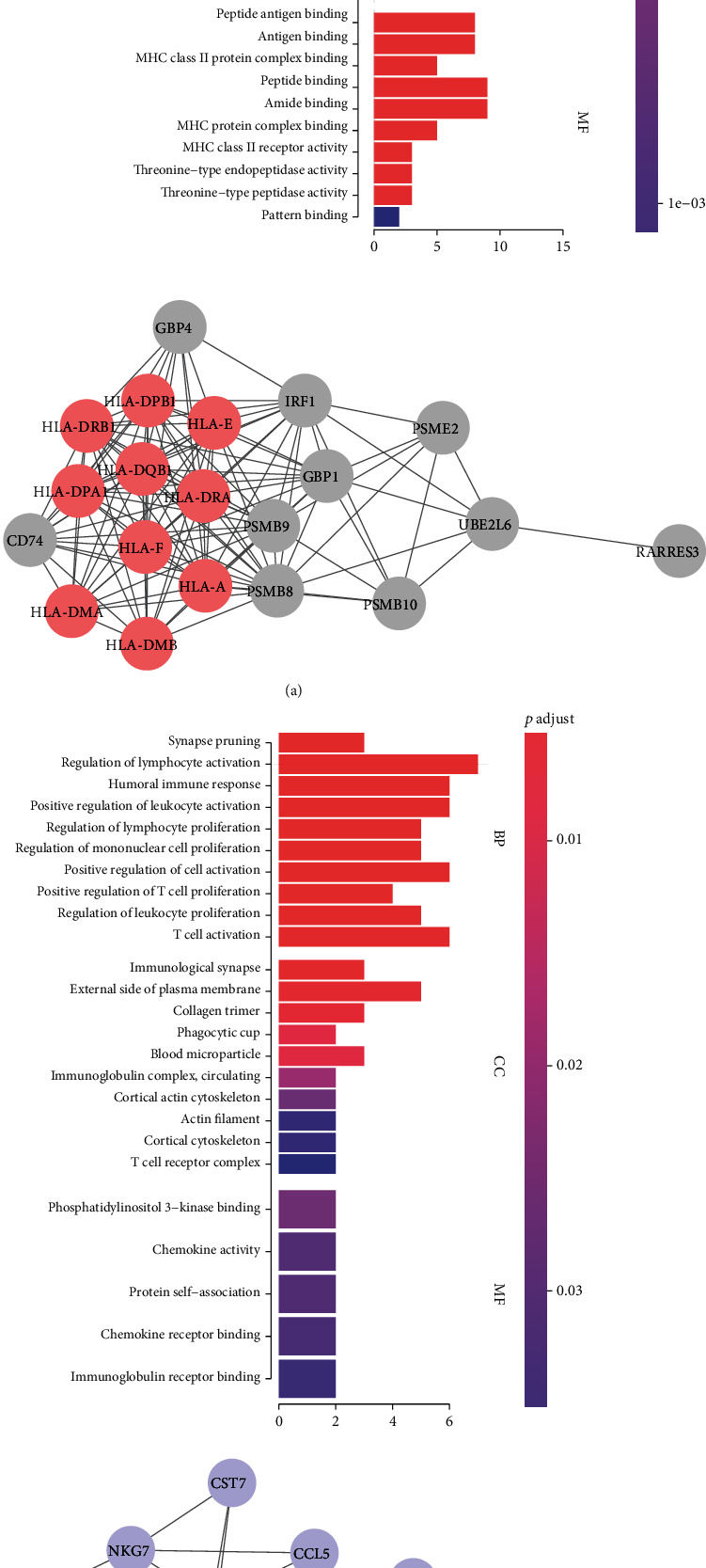
Functional analysis and protein-protein network of grey60 and magenta modules. (a) Grey60 module. (b) Magenta module.

We determined the top 20 CD8+ T lymphocyte proportions positively coexpressing mRNA in TCGA-LIHC grey60 and magenta modules (Tables 1 and 2). The 20 CD8+ T lymphocyte proportions positively coexpressing mRNA in the magenta module were most significantly enriched in antigen processing and presentation. The 20 CD8+ T lymphocyte proportions positively coexpressing mRNA in the grey60 module were most significantly enriched in regulating lymphocyte activation, suggesting that these biological processes might promote CD8+ T lymphocyte infiltration in the liver hepatocellular cancer microenvironment (Figures 3(a) and 3(b)). The protein-protein interaction network of yellow and green modules is shown in Figure 3.

### 3.4. Clinical Phenotype and Immune Phenotype Correlation of Coexpression Genes

**Figure 4:**
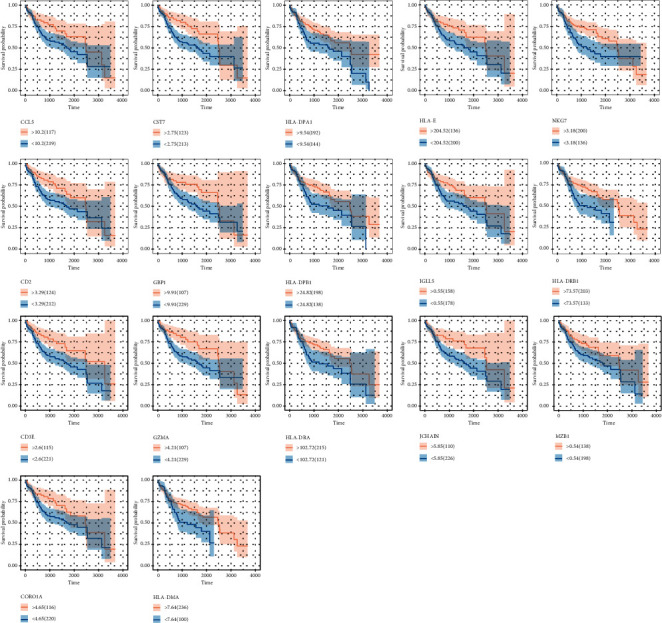
The survival analysis of genes in magenta and grey60 modules. The grey60 module genes acted as prognostic protective genes.

**Figure 5:**
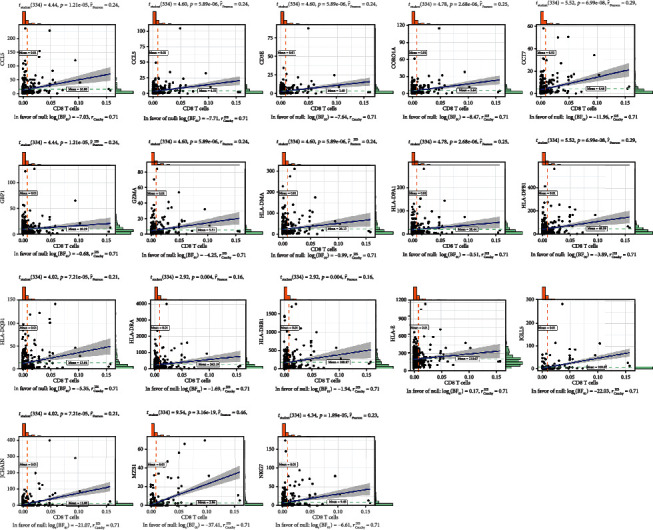
The Pearson correlation test among genes in magenta and grey60 modules with CD8+ T cell proportions.

**Figure 6:**
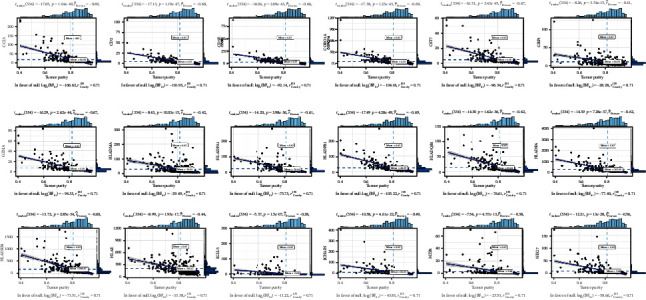
The Pearson correlation test among genes in magenta and grey60 modules with tumor purity.

To determine the outcome status correlation of these coexpression genes, we performed survival analysis. The patients in low expression groups for CCL5 (TCGA: *p* = 0.03), CST7 (TCGA: *p* = 0.005), HLA-DPA1 (TCGA: *p* = 0.012), HLA-E (TCGA: *p* = 0.042), NKG7 (TCGA: *p* = 0.007), CD2 (TCGA: *p* = 0.013), GBP1 (TCGA: *p* = 0.037), HLA-DPB1 (TCGA: *p* = 0.034), IGLL5 (TCGA: *p* = 0.029), HLA-DRB1 (TCGA: *p* = 0.014), CD3E (TCGA: *p* = 0.004), GZMA (TCGA: *p* = 0.006), HLA-DRA (TCGA: *p* = 0.036), JCHAIN (TCGA: *p* = 0.012), MZB1 (TCGA: *p* = 0.036), CORO1A (TCGA: *p* = 0.034), and HLA-DMA (TCGA: *p* = 0.04) showed survival risk against high expression groups (Figure 4). These results suggest that these coexpression genes in grey60 and magenta modules protect against liver hepatocellular cancer. Next, we found that these factors were positively correlated with CD8+ T lymphocytes (Figure 5) and negatively correlated with tumor purity (Figure 6).

### 3.5. Cox Regression Hazard Model of CD8+ T Lymphocyte Coexpression Genes

A CD8+ T lymphocyte coexpression gene Cox regression hazard model was conducted based on these liver hepatocellular outcome protective factors.

(1) $$\begin{array}{c} \text{Risk}=0.005*\text{C}1\text{QC}+0.036*\text{CD}3\text{D}-0.123*\text{GZMA}+0.012*\text{PSMB}9. \end{array}$$

**Figure 7:**
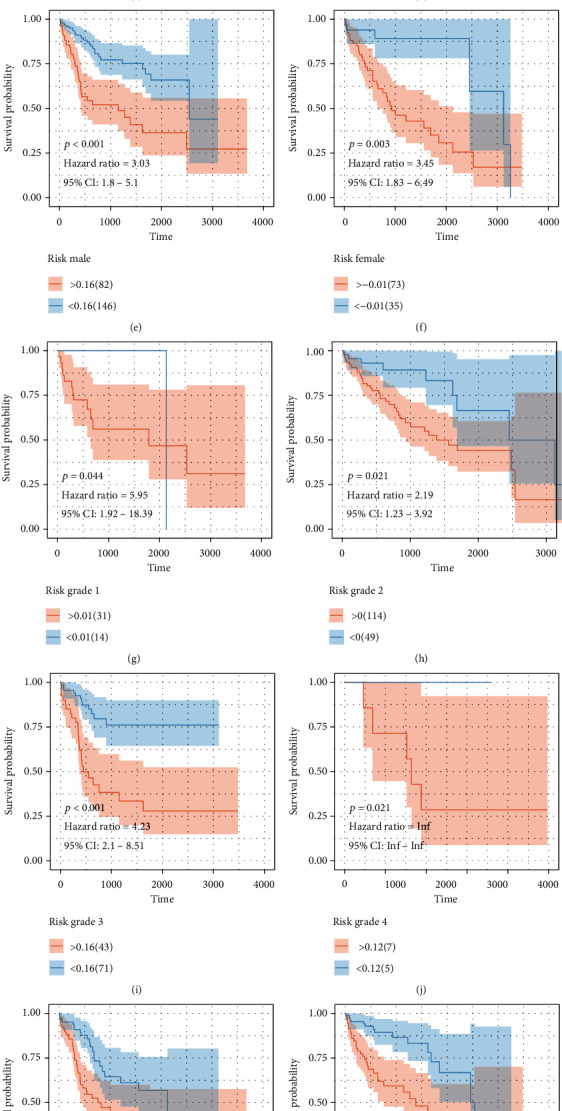
Cox regression analysis based on these CD8+ T cell-related genes. (a) The patients in the higher risk group showed worse outcome levels. (b) The receiver operating characteristic curve of the risk score in TCGA-LIHC cohort. (c–n) The subgroup survival analysis of CD8+ T cell-related gene risk scores.

The samples in high-risk samples for liver hepatocellular cancer patients (TCGA: *p* < 0.001; HR = 23) (Figure 7) showed survival risk against low-risk groups, with AUC = 0.67 (Figure 7). The risk score was evaluated in various subgroups, including age, gender, stage, tumor purity, and tumor mutation burden. The results were significant in these subgroups.

### 3.6. GSEA

**Figure 8:**
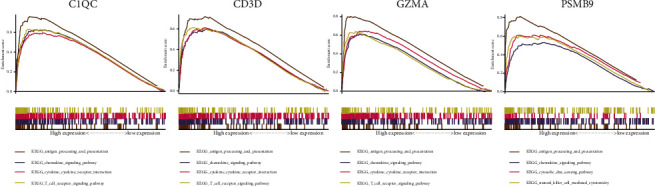
GSEA.

Antigen processing and presentation, the chemokine signaling pathway, the cytokine-cytokine receptor interaction pathway, and the T cell receptor signaling pathway were related to the high expression group in C1QC, CD3D, GZMA, and PSMB9 (Figure 8).

### 3.7. Tissue Verification and Single-Cell Markers

**Figure 9:**
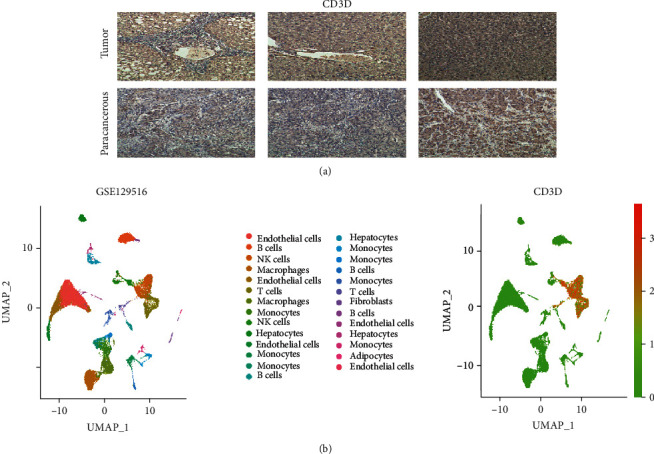
(a) Immunohistochemical analysis in tumor and paracarcinoma tissues from China Medical University clinical samples. (b) Single-cell cohort validation in GSE129516.

Immunohistochemical analysis of the protein expression levels of CD3D in and around carcinomas was performed in the liver cancer cohorts of the First Affiliated Hospital of China Medical University. The results showed that CD3D had lower staining intensity (Figure 9(a)). We also performed UMAP dimensional reduction clustering in the data of a single cell of liver cancer in GEO. After annotating the subsets with “SingleR,” we obtained the subsets containing T cell macrophages and other cells. We found that the expression content of CD3D was relatively high in the T cell subsets, which confirmed our previous conclusion in TCGA-LIHC (Figure 9(b)).

We analyzed the relationship between CD3D and currently known gene sets in the tumor microenvironment and verified the expression association between CD3D and CD8A in two other cohorts, GSE29721 [22] and GSE121248 [23]. We also marked the distribution of different T cell subtypes in single-cell cohorts, and the results showed that CD3D was more strongly associated with CD8A and less with CD4+ T lymphocytes (Supplementary Figure 1).

## 4. Discussion

Immunotherapy is a late-stage cancer treatment strategy widely used in clinical practice; nevertheless, there are many unsatisfactory results, including the low success rate of immunotherapy, complications associated with immunotherapy, and the super progression of immunotherapy. Studies have shown that the low success rate of immunotherapy is related to the low infiltration of CD8+ T lymphocytes, the weakening of the antigen presentation process, and the reduction of PD-1 expression. In this study, we explored the coexpression network that promotes the infiltration of CD8+ T lymphocytes in liver cancer by combining computational biology and experiments. The factors in the coexpression network are considered to have similar biological functions in organisms. Therefore, mining these coexpression factors helps us understand the biological processes most closely related to the infiltration of CD8+ T lymphocytes in liver cancer.

The grey60 module was the most relevant coexpression module for CD8+ T lymphocytes. The factors in this module are related to the regulation of T cell proliferation. To date, we have demonstrated that these factors are related to the infiltration of CD8+ T lymphocytes at the sequencing and functional levels. Based on our inferences, we believe that these factors may improve outcomes by increasing the infiltration of CD8+ T lymphocytes. Then, we conducted survival analysis on these factors and successfully constructed a prognostic risk score for liver cancer based on CD8+ T lymphocyte coexpression factors. The differences in protein levels of C1QC, CD3D, GZMA, and PSMB9 in different liver cancer stages were determined using immunohistochemistry.

*C1QC* encodes the complement C1q C chain, which associates with C1r and C1s to yield the first component of the serum complement system [24, 25]. C1q is composed of 18 polypeptide chains which include six A-chains, six B-chains, and six C-chains. Each chain contains an N-terminal collagen-like region and a C-terminal C1q globular domain. The protein encoded by CD3D is part of the T cell receptor/CD3 complex (TCR/CD3 complex) and is involved in T cell development and signal transduction [26, 27]. The encoded membrane protein represents the delta subunit of the CD3 complex. Along with four other CD3 subunits, the encoded membrane protein binds either TCR alpha/beta or TCR gamma/delta to form the TCR/CD3 complex on the surface of T cells [28]. In this study, transcriptome, histological, and single-cell cohorts were used to demonstrate the importance of C1QC and CD3D in CD8+ T lymphocytes.

This article has some limitations. We used TCGA and liver cancer tissues from China Medical University to conduct a joint analysis. More external cohorts still need to be cross-validated. Due to the limited computing power of computers, we only included factors with variances in the top 25% when performing WGCNA. This may cause some false-negative results. More advanced computers need to be used to repeat this screening method. Although we hypothesize that these factors can improve the therapeutic effect of liver cancer immunotherapy by promoting the infiltration of CD8+ T lymphocytes, due to the lack of immunotherapy efficacy evaluation in the follow-up data of TCGA, we only explored the passage of these coexpression factors in liver cancer. More cohorts with immune follow-up data need to be added to promote the infiltration of CD8+ T lymphocytes and improve the outcome of patients.

In summary, we constructed a prognostic risk scoring model for liver cancer based on a CD8+ T lymphocyte content coexpression molecular network, determined that the factors in the risk scoring model can be used as independent prognostic factors for liver cancer, and determined the levels of these factors at the mRNA and protein levels. These prognosis-related CD8+ T lymphocyte coexpression factors and their related biological functions may provide new directions for improving the efficacy of immunotherapy.

## Figures and Tables

**Table 1:** The module and gene significance for CD8+ T cell-related genes in the grey60 module.

ID	Module color	GS.T.cells.CD8	P.GS.T.cells.CD8
PSMB9	Grey60	0.42727727	5.76*e* − 15
PSMB10	Grey60	0.3960461	6.76*e* − 13
PSMB8	Grey60	0.392423292	1.14*e* − 12
HLA-E	Grey60	0.392387821	1.14*e* − 12
GBP4	Grey60	0.38525836	3.13*e* − 12
RARRES3	Grey60	0.379808931	6.66*e* − 12
CD74	Grey60	0.379010979	7.43*e* − 12
HLA-DQB1	Grey60	0.378859742	7.58*e* − 12
PSME2	Grey60	0.378610599	7.84*e* − 12
GBP1	Grey60	0.377173717	9.54*e* − 12
HLA-DPB1	Grey60	0.376684402	1.02*e* − 11
HLA-DRB1	Grey60	0.368162706	3.18*e* − 11
HLA-A	Grey60	0.359477552	9.81*e* − 11
IRF1	Grey60	0.345346667	5.71*e* − 10
HLA-DMA	Grey60	0.337351935	1.49*e* − 09
HLA-F	Grey60	0.334500299	2.08*e* − 09
UBE2L6	Grey60	0.333949723	2.22*e* − 09
HLA-DRA	Grey60	0.328624957	4.11*e* − 09
HLA-DPA1	Grey60	0.318999761	1.21*e* − 08
HLA-DMB	Grey60	0.315091809	1.86*e* − 08

GS: gene significance.

**Table 2:** The module and gene significance for CD8+ T cell-related genes in the magenta module.

ID	Module color	GS.T.cells.CD8	P.GS.T.cells.CD8
NKG7	Magenta	0.626	1.14*e* − 34
CST7	Magenta	0.592	3.00*e* − 30
GZMA	Magenta	0.544	5.89*e* − 25
CCL5	Magenta	0.523	7.31*e* − 23
CXCL9	Magenta	0.470	3.59*e* − 18
HCST	Magenta	0.463	1.22*e* − 17
CD3D	Magenta	0.421	1.35*e* − 14
CD2	Magenta	0.413	5.29*e* − 14
CALHM6	Magenta	0.406	1.55*e* − 13
GMFG	Magenta	0.405	1.57*e* − 13
MZB1	Magenta	0.397	5.69*e* − 13
C1QA	Magenta	0.395	7.18*e* − 13
PIM2	Magenta	0.389	1.82*e* − 12
CD3E	Magenta	0.384	3.42*e* − 12
C1QB	Magenta	0.375	1.15*e* − 11
IGLL5	Magenta	0.367	3.32*e* − 11
CORO1A	Magenta	0.362	6.86*e* − 11
AIF1	Magenta	0.355	1.59*e* − 10
C1QC	Magenta	0.341	9.21*e* − 10
JCHAIN	Magenta	0.333	2.36*e* − 09

GS: gene significance.

## Data Availability

The datasets TCGA-LIHC for this study can be found at http://cancergenome.nih.gov/. The datasets GSE129516, GSE29721, and GSE121248 in this study can be found at http://www.ncbi.nlm.nih.gov/geo/.

## Supplementary Materials

Supplementary Materials Supplementary Figure 1: CD3D immune environment analysis and verification. (A) CD3D expression positively correlates to the intensity immune response. (B) CD3D expression positively correlates to the CD8A in GSE29721 and GSE121248. (C) The distribution of CD8A and CD4.
