# Supplementary figures and images for: Identification of CD8+ T Cell-Related Genes: Correlations with Immune Phenotypes and Outcomes of Liver Cancer
Source: J Immunol Res. 2021 Jun 2;2021:9960905. doi: 10.1155/2021/9960905 (PMC8192185; doi:10.1155/2021/9960905)

A

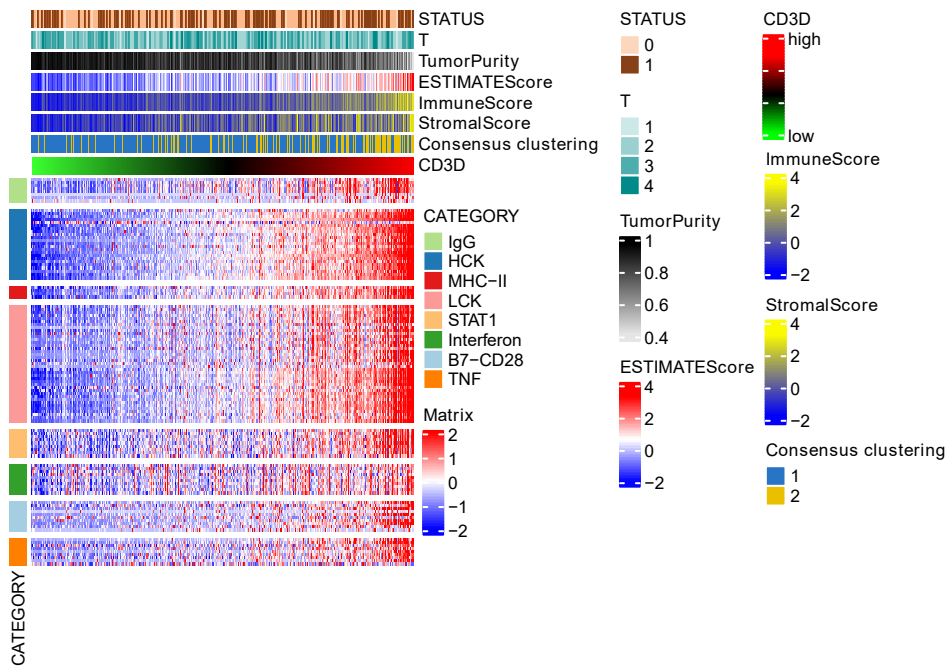

B

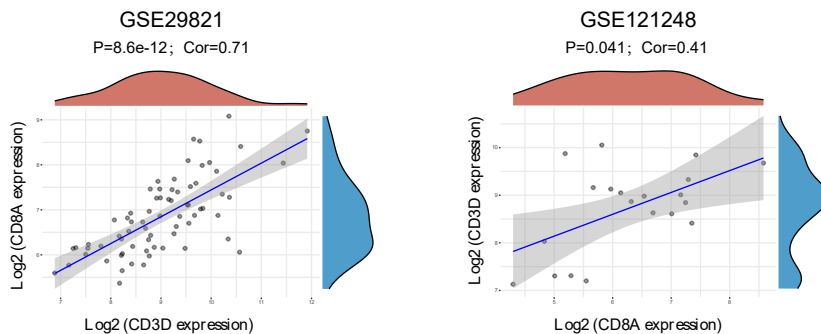

C

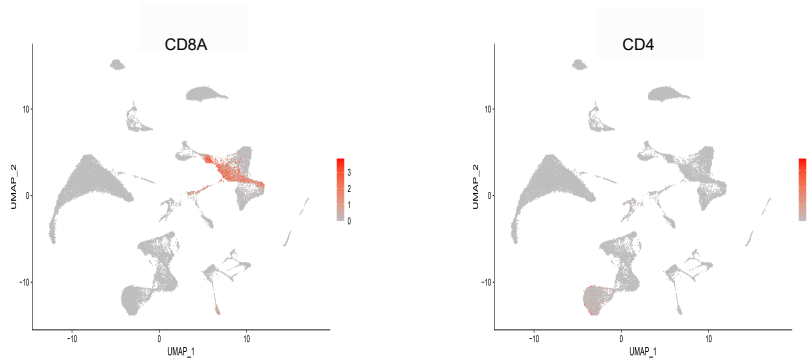

Supplement: Supplementary Materials — Supplementary Figure 1: CD3D immune environment analysis and verification. (A) CD3D expression positively correlates to the intensity immune response. (B) CD3D expression positively correlates to the CD8A in GSE29721 and GSE121248. (C) The distribution of CD8A and CD4. [file 9960905.f1.pdf]
